# Association between pancreatic ectopic fat deposition and QCT-assessed bone mass status in patients with chronic kidney disease: a cross-sectional study

**DOI:** 10.1080/0886022X.2026.2749918

**Published:** 2026-11-02

**Authors:** Jiahui Yang, Ruxia Wang, Guozhong Xue, Jinhui Wei, Lin Wang

**Affiliations:** ^a^ The First Clinical Medical College, Gansu University of Chinese Medicine, Lanzhou, China; ^b^ Department of Radiology, Affiliated Hospital of Gansu University of Chinese Medicine, Lanzhou, China; ^c^ Department of Nephrology, Affiliated Hospital of Gansu University of Chinese Medicine, Lanzhou, China

**Keywords:** Chronic kidney disease, quantitative computed tomography, volumetric bone mineral density, pancreatic fat fraction, body composition

## Abstract

**Background:**

Patients with chronic kidney disease (CKD) are at increased risk of bone abnormalities. Beyond disturbances in mineral metabolism, ectopic fat deposition may also be associated with skeletal abnormalities in CKD. However, the relationship between pancreatic fat and bone mass status remains unclear. This study aimed to evaluate the association between mean pancreatic fat fraction and quantitative computed tomography (QCT)-assessed bone mass status in patients with CKD.

**Methods:**

This retrospective cross-sectional study included 327 patients with CKD. Vertebral volumetric bone mineral density was measured using QCT to classify bone mass status as normal bone mass, osteopenia, or osteoporosis. Mean pancreatic fat fraction was quantified from CT images. Ordinal logistic regression, restricted cubic spline (RCS), subgroup analyses, and sensitivity analyses were performed to evaluate its association with bone mass status.

**Results:**

Mean pancreatic fat fraction was highest in patients with osteoporosis (*p* < 0.001). In the fully adjusted model, each 5-percentage-point increase in mean pancreatic fat fraction was associated with higher odds of a more severe bone mass category (OR = 1.481, 95% CI: 1.152–1.905, *p* = 0.002). RCS analysis showed a significant overall association (*p* = 0.009), with no evidence of nonlinearity (*p* = 0.290). No statistically significant interactions were detected for sex, age, diabetes status, or CKD stage.

**Conclusion:**

A higher mean pancreatic fat fraction was associated with more severe bone mass categories in patients with CKD after adjustment for the measured covariates. QCT-based pancreatic fat assessment may provide additional information on metabolic features associated with bone mass abnormalities in CKD.

## Introduction

Chronic kidney disease (CKD) is a major global public health concern, with a high prevalence and substantial disease burden worldwide [1,2]. As kidney function declines, patients with CKD may develop disturbances in mineral and bone metabolism, resulting in reduced bone mass and an increased risk of osteoporosis and fractures [3]. CKD–mineral and bone disorder (CKD-MBD) encompasses a complex spectrum of pathophysiological alterations, including impaired phosphate metabolism, elevated fibroblast growth factor 23 levels, vitamin D deficiency, and secondary hyperparathyroidism [3,4]. However, the mechanisms underlying abnormal bone mass in patients with CKD are complex and extend beyond traditional mineral metabolism disturbances. Emerging evidence suggests that alterations in body composition may also be associated with skeletal abnormalities in patients with CKD [5].

The relationship between adipose tissue and bone metabolism extends beyond total adiposity, with increasing attention being paid to the effects of fat distribution and ectopic fat deposition on skeletal health. Excess lipids can accumulate abnormally in non-adipose tissues, such as the liver, pancreas, and skeletal muscle, resulting in distinct ectopic fat phenotypes that are associated with metabolic disturbances, inflammatory responses, and lipotoxicity [6]. Pancreatic fat deposition represents an important manifestation of ectopic adiposity and has been closely linked to obesity, insulin resistance, type 2 diabetes mellitus, and metabolic syndrome [7]. In addition, abnormal fat distribution and ectopic fat accumulation at different anatomical sites have also been associated with lower bone mass [8,9]. However, studies investigating the relationship between pancreatic fat deposition and bone mass status remain limited. Existing evidence has primarily focused on individuals with diabetes or obesity, and whether pancreatic fat is associated with bone mass status in patients with CKD remains unclear.

Moreover, recent studies have suggested that pancreatic fat deposition is associated with impaired kidney function and an increased risk of CKD development [10]. Patients with CKD frequently present with concurrent renal dysfunction, metabolic disturbances, and alterations in body composition, which may influence the relationship between pancreatic fat and bone mass status. Quantitative computed tomography (QCT) enables the quantitative assessment of vertebral volumetric bone mineral density (vBMD) [11]. In addition, CT-based analysis allows for the quantitative evaluation of body composition and pancreatic fat content. Previous studies have shown a good correlation between pancreatic fat measurements obtained using QCT and magnetic resonance imaging (MRI) [12]. Therefore, this study aimed to investigate the association between pancreatic fat fraction and bone mass status in patients with CKD using abdominal CT and QCT-based body composition analysis. Furthermore, we explored the dose–response relationship and assessed the consistency of this association across different clinical subgroups.

## Method

### Study population

This retrospective study was approved by the Ethics Committee of the Affiliated Hospital of Gansu University of Chinese Medicine (Approval No. 2026015), and the requirement for informed consent was waived. Patients with CKD who were hospitalized in the Department of Nephrology at the Affiliated Hospital of Gansu University of Chinese Medicine between November 2023 and July 2026 and had undergone abdominal CT were retrospectively identified. After screening according to the predefined inclusion and exclusion criteria, 327 patients were included in the final analysis. Given the retrospective design, no *a priori* sample-size calculation was performed; instead, all eligible patients identified during the study period were included. The patient selection process is shown in Figure 1.

**Figure 1.**
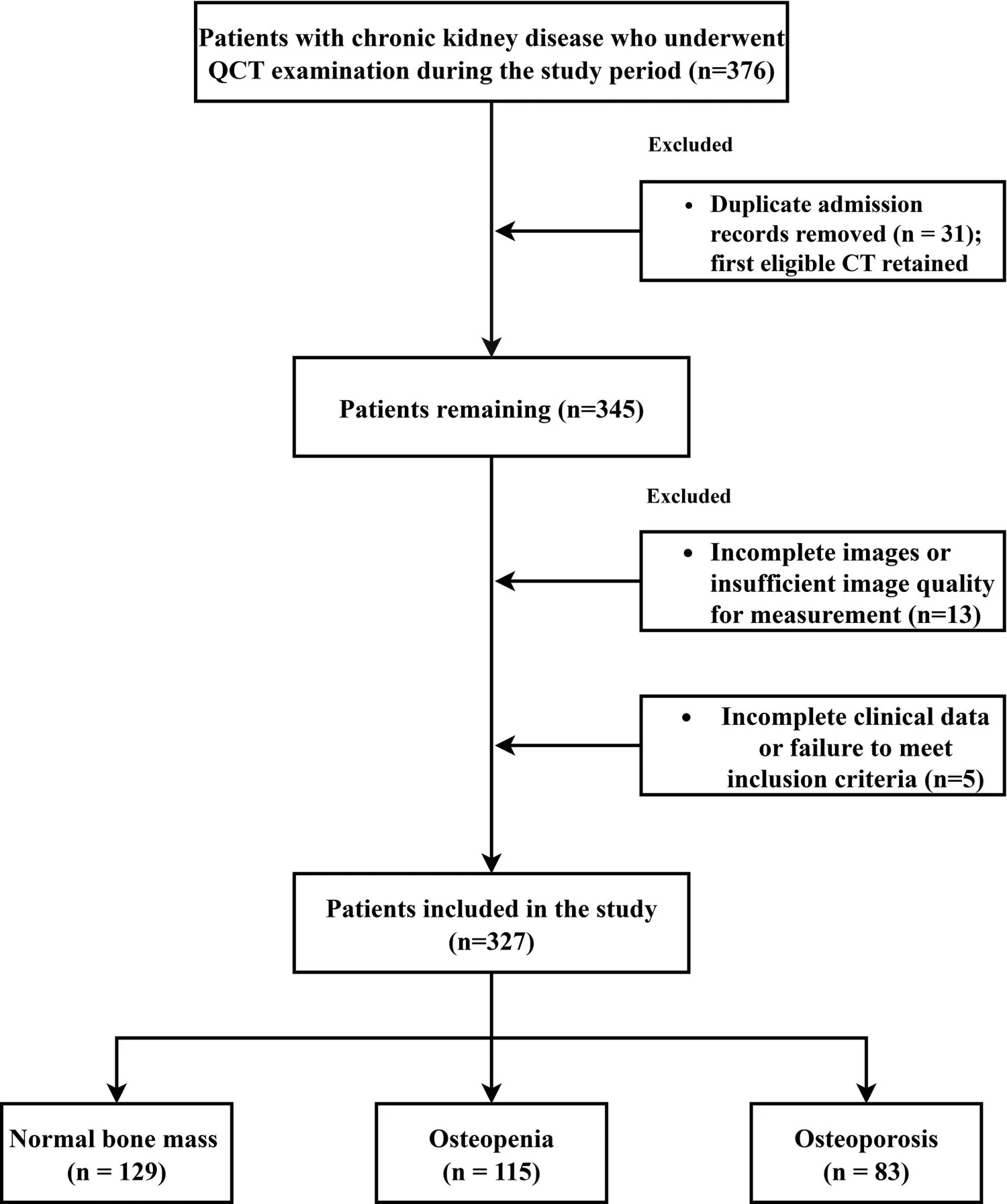
Flowchart of patient selection.

The inclusion criteria were as follows: (1) diagnosis of CKD according to the Kidney Disease: Improving Global Outcomes (KDIGO) criteria; (2) age ≥18 years; (3) abdominal CT performed for clinical indications during the study period, with complete CT datasets available for QCT analysis; (4) CT image quality sufficient for quantitative assessment of pancreatic fat fraction, L1 and L2 vertebral vBMD, and L3 skeletal muscle parameters; and (5) availability of the clinical and laboratory data required for the study.

The exclusion criteria were as follows: (1) a history of pancreatic surgery or pancreatic disease that could substantially affect pancreatic fat quantification; (2) focal abnormalities of the L1–L3 vertebrae that could interfere with quantitative measurements; (3) other diseases known to substantially affect bone metabolism; and (4) long-term systemic glucocorticoid therapy or use of other medications known to affect bone metabolism. For patients with multiple eligible CT examinations during the study period, only the first eligible examination was included to avoid duplicate inclusion.

### Data collection

Demographic characteristics, clinical data, and laboratory measurements were obtained from the electronic medical record system. Demographic and clinical variables included sex, age, body mass index (BMI), hypertension, diabetes mellitus, dialysis status, and CKD stage. Laboratory measurements included hemoglobin (Hb), serum creatinine (Scr), uric acid (UA), urea, albumin (ALB), serum calcium (Ca), and serum phosphorus (P), triglycerides (TG), total cholesterol (CHOL), low-density lipoprotein cholesterol (LDL-C), and high-density lipoprotein cholesterol (HDL-C). Laboratory results obtained within one week of the abdominal CT examination were collected, with the measurement closest to the CT examination selected for analysis. For the 51 patients receiving dialysis, the selected blood samples were collected on the day of admission or the following day, before any dialysis session during that hospitalization. All laboratory tests were performed by the Department of Laboratory Medicine at the Affiliated Hospital of Gansu University of Chinese Medicine.

Estimated glomerular filtration rate (eGFR) was calculated using the 2021 Chronic Kidney Disease Epidemiology Collaboration (CKD-EPI) creatinine equation based on age, sex, and Scr [13]. CKD G categories were defined according to the KDIGO guidelines based on eGFR; G3a and G3b were combined into a single G3 category for statistical analyses [14].

### CT image acquisition and QCT analysis

All patients underwent abdominal CT scanning in the supine position with both arms positioned above the head. Patients were instructed to maintain quiet breathing during image acquisition. CT examinations were performed using a third-generation dual-source CT scanner (Siemens Healthineers, Germany). A Mindways QCT calibration phantom (Mindways Software, Inc., Austin, TX, USA) was placed beneath the patient during scanning. The scanning parameters were as follows: tube voltage, 120 kV; tube current, 125 mAs; pitch, 1.2; and collimation width, 96 × 0.6 mm. Automatic tube current modulation was applied using CARE Dose4D (Siemens Healthineers). CT images were reconstructed with a slice thickness of 1 mm and a matrix size of 512 × 512 pixels. All CT images were transferred to the QCT Pro workstation (version 6.1.0; Mindways Software, Inc., Austin, TX, USA) for quantitative assessment of bone mineral density and body composition. All CT measurements were performed by a single radiologist who was blinded to the patients’ bone mass classification.

Vertebral vBMD was measured using the QCT Pro workstation. The mean vBMD value of the L1 and L2 vertebral bodies was used for subsequent analyses. According to the lumbar spine QCT-based bone mass classification criteria [15], patients were categorized into three groups: normal bone mass (vBMD >120 mg/cm³), osteopenia (vBMD 80–120 mg/cm³), and osteoporosis (vBMD <80 mg/cm³). For vBMD measurements, the region of interest (ROI) was placed in the central trabecular bone area of each vertebral body, avoiding the cortical bone and basivertebral venous plexus. The ROI area was maintained between 300 and 350 mm^2^. The Mindways QCT calibration phantom was used for quality control throughout the measurement process.

Body composition analysis was performed at the level of the mid-L3 vertebral body using a CT attenuation threshold-based semi-automated segmentation method. The CT attenuation thresholds were set at −190 to −30 HU for adipose tissue and −29 to 150 HU for skeletal muscle [16]. First, tissues within the predefined CT attenuation thresholds were automatically segmented. The intra-abdominal region was then manually delineated and excluded from the automatically segmented region. The remaining skeletal muscle area, including the abdominal wall, paraspinal, and psoas muscles, was defined as the L3 skeletal muscle area (L3-SMA) (Figure 2A, B). The L3 skeletal muscle index (L3-SMI) was calculated by normalizing L3-SMA to height squared: L3-SMI = L3-SMA (cm^2^)/height^2^ (m^2^). At the L3 level, intermuscular adipose tissue (IMAT), subcutaneous adipose tissue (SAT), and visceral adipose tissue (VAT) areas were also measured. The visceral-to-subcutaneous fat ratio (V/S ratio) was calculated as the ratio of VAT area to SAT area.

**Figure 2.**
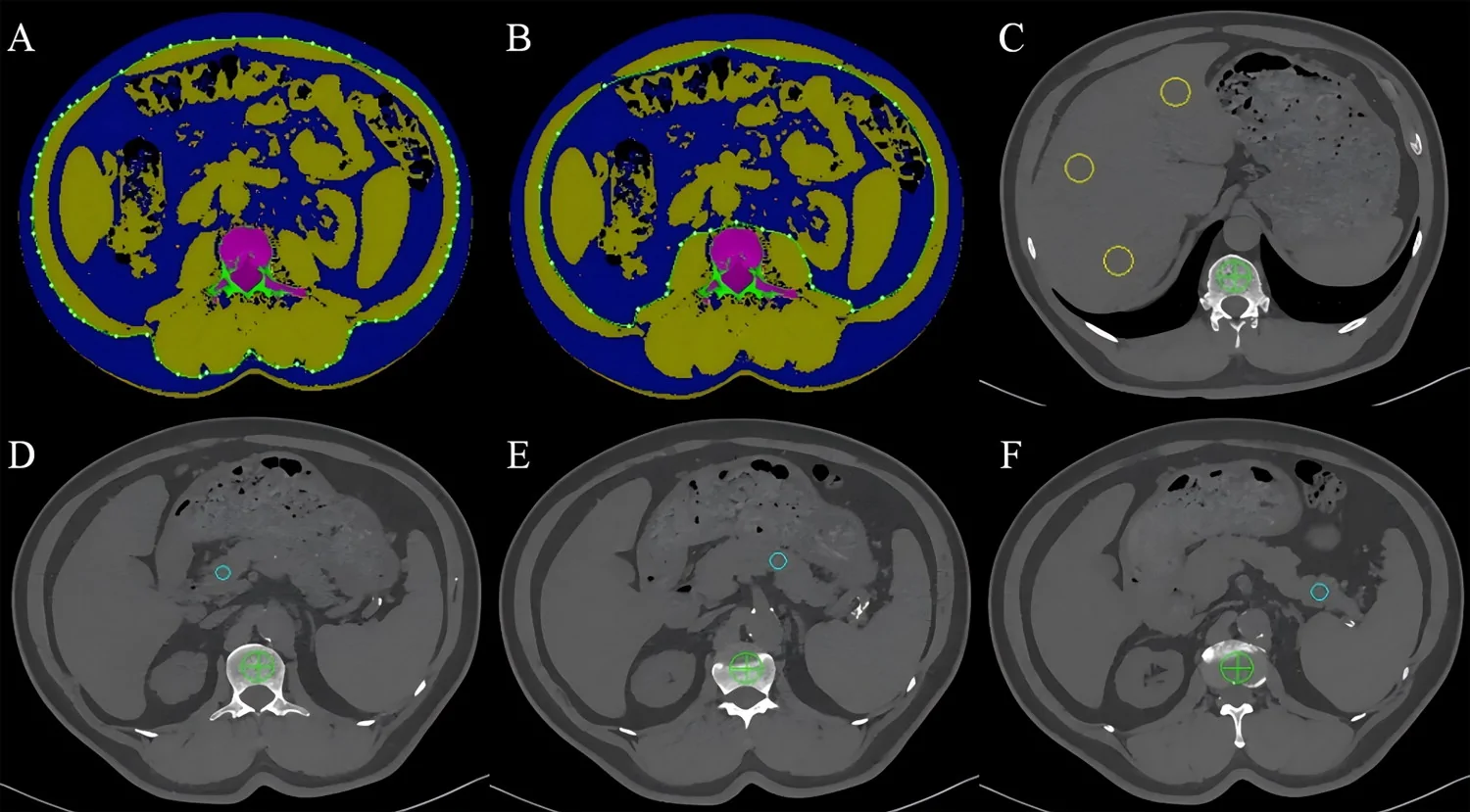
Representative images of QCT-based body composition analysis and fat quantification. (A, B) Body composition analysis at the level of the third lumbar vertebra (L3). (A) Automatic segmentation based on predefined CT attenuation thresholds, including the abdominal wall, paraspinal, and psoas muscles and intra-abdominal contents; (B) manual delineation of the intra-abdominal region. The L3 skeletal muscle area (L3-SMA) was obtained by subtracting the intra-abdominal region from the automatically segmented region. (C) Measurement of liver fat fraction. ROIs were placed in the left lobe, right anterior lobe, and right posterior lobe of the liver, and the fat fractions from the three ROIs were averaged to obtain the mean liver fat fraction. (D–F) Measurement of pancreatic fat fraction. ROIs were placed in the pancreatic head (D), body (E), and tail (F), and the fat fractions from the three regions were averaged to obtain the mean pancreatic fat fraction.

For hepatic fat quantification, ROIs were placed in the left lobe, right anterior lobe, and right posterior lobe of the liver on an axial image obtained approximately at the level of the left portal vein. Each ROI covered an area of approximately 300 mm^2^ and was positioned to avoid intrahepatic large vessels, bile ducts, and obvious imaging artifacts (Figure 2C). The fat fraction within each ROI was recorded, and the mean value of the three ROIs was calculated as the mean liver fat fraction.

Pancreatic fat fraction was assessed using an ROI-based QCT approach consistent with previously published methods [7,12]. Circular ROIs of approximately 100–150 mm^2^ were placed in the pancreatic head, body, and tail, carefully avoiding visible vessels, the pancreatic duct, surrounding adipose tissue, and imaging artifacts (Figure 2D–F). The mean of the three regional measurements was calculated as the mean pancreatic fat fraction.

To evaluate the interobserver reproducibility of QCT parameter measurements, 40 patients were randomly selected for repeated measurements. A second radiologist independently performed the measurements following the same protocol and was blinded to the results of the first radiologist. Interobserver reproducibility was assessed using the intraclass correlation coefficient (ICC) with 95% confidence intervals (CIs).

### Statistical analysis

Statistical analyses were performed using R software (version 4.6.1). The normality of continuous variables was assessed using the Shapiro–Wilk test and Q–Q plots. Normally distributed continuous variables were presented as mean ± standard deviation (SD), whereas non-normally distributed variables were presented as median (interquartile range [IQR]). Comparisons of continuous variables among the three groups were performed using one-way analysis of variance (ANOVA) or the Kruskal–Wallis H test, as appropriate. Categorical variables were presented as n (%), and comparisons among groups were performed using Pearson’s chi-square test or Fisher’s exact test, as appropriate.

Bone mass status defined by QCT (normal bone mass, osteopenia, and osteoporosis) was considered an ordinal outcome variable. Ordinal logistic regression models were used to evaluate the association between mean pancreatic fat fraction and the odds of being in a more severe bone mass category. Mean pancreatic fat fraction was entered into the models as a continuous variable, and the effect estimate was expressed per 5-percentage-point increase using odds ratios (ORs) and 95% CIs. Covariates in the primary models were selected based on clinical relevance and previously reported associations with ectopic fat deposition and/or bone health in patients with CKD. The crude model was unadjusted; Model 1 was adjusted for age and sex; Model 2 was additionally adjusted for BMI, eGFR, diabetes mellitus, and hypertension; and Model 3 was further adjusted for L3-SMI and was considered the fully adjusted model. The correlation between mean pancreatic fat fraction and L3-SMI was assessed using Spearman’s rank correlation. Multicollinearity among covariates included in the multivariable models was assessed using variance inflation factors (VIFs). The proportional odds assumption was assessed using the Brant test to verify the validity of the ordinal logistic regression models.

Restricted cubic spline (RCS) analysis was further performed to evaluate the dose–response relationship between mean pancreatic fat fraction and the odds of being in a more severe bone mass category. The overall association and departure from linearity were assessed using the P values for overall association and nonlinearity, respectively.

To assess the consistency of the association between mean pancreatic fat fraction and bone mass status across clinical subgroups, subgroup analyses were performed according to sex, age (<60 and ≥60 years), diabetes status, and CKD stage (G1–G2 and G3–G5). Subgroup analyses were adjusted for the covariates included in the fully adjusted model, except for the variable used for stratification. In the CKD stage subgroup analysis, eGFR was not additionally adjusted. Potential effect modification was evaluated by introducing interaction terms between pancreatic fat fraction and each stratification variable into the regression models.

To assess the potential influence of dialysis status, a sensitivity analysis was performed after excluding patients receiving dialysis. Baseline characteristics and the association between mean pancreatic fat fraction and bone mass status were reevaluated in the non-dialysis population, with the ordinal logistic regression models constructed using the same covariate-adjustment strategy as in the primary analysis.

All statistical tests were two-sided, and a P value <0.05 was considered statistically significant.

## Results

### Clinical and imaging characteristics according to bone mass status

A total of 327 patients with CKD were included in the final analysis, including 129 patients with normal bone mass, 115 with osteopenia, and 83 with osteoporosis. Significant differences were observed among the three groups in sex distribution, age, prevalence of diabetes mellitus, and CKD stage (all *p* < 0.05), whereas no significant differences were found in BMI, prevalence of hypertension, or dialysis status (all *p* > 0.05). Regarding laboratory measurements, Hb and eGFR levels differed significantly among the three groups (both *p* < 0.05). No significant differences were observed in urea, ALB, Scr, UA, serum calcium, or serum phosphorus levels (all *p* > 0.05).

For CT-derived body composition parameters, significant differences were observed among the three groups in L3-SMA, L3-SMI, and IMAT (all *p* < 0.001). Patients with osteoporosis had the lowest L3-SMA and L3-SMI values and the highest IMAT area among the three groups. In contrast, SAT, VAT, and the V/S ratio showed no significant differences among the three groups (all *p* > 0.05). Furthermore, mean pancreatic fat fraction differed significantly among the three groups (*p* < 0.001), with the highest value observed in the osteoporosis group. No significant difference was found in mean liver fat fraction among the three groups (*p* = 0.711). Detailed results are presented in Table 1.

**Table 1.** Clinical and imaging characteristics of CKD patients according to bone mass status.

Characteristics	Overall (*N* = 327)	Normal bone mass (*N* = 129)	Osteopenia (*N* = 115)	Osteoporosis (*N* = 83)	*P* value
Demographic and clinical characteristics					
Sex, n (%)					<0.001
Male	208 (63.6)	92 (71.3)	78 (67.8)	38 (45.8)	
Female	119 (36.4)	37 (28.7)	37 (32.2)	45 (54.2)	
Age (years)	57.90 ± 13.31	47.81 ± 12.09	61.45 ± 9.02	68.67 ± 8.25	<0.001
BMI (kg/m²)	24.03 (21.88, 26.12)	24.21 (21.97, 26.83)	23.99 (22.21, 26.30)	24.03 (21.46, 25.69)	0.314
Hypertension, n (%)					0.053
No	109 (33.3)	50 (38.8)	40 (34.8)	19 (22.9)	
Yes	218 (66.7)	79 (61.2)	75 (65.2)	64 (77.1)	
Diabetes mellitus, n (%)					<0.001
No	213 (65.1)	102 (79.1)	63 (54.8)	48 (57.8)	
Yes	114 (34.9)	27 (20.9)	52 (45.2)	35 (42.2)	
Dialysis status, n (%)					0.206
No	276 (84.4)	108 (83.7)	102 (88.7)	66 (79.5)	
Yes	51 (15.6)	21 (16.3)	13 (11.3)	17 (20.5)	
CKD stage, n (%)					0.004
G1	81 (24.8)	44 (34.1)	28 (24.3)	9 (10.8)	
G2	66 (20.2)	21 (16.3)	28 (24.3)	17 (20.5)	
G3	49 (15.0)	15 (11.6)	21 (18.3)	13 (15.7)	
G4	38 (11.6)	10 (7.8)	13 (11.3)	15 (18.1)	
G5	93 (28.4)	39 (30.2)	25 (21.7)	29 (34.9)	
Laboratory parameters					
Hemoglobin (g/L)	128.29 ± 29.87	131.95 ± 32.03	131.84 ± 28.09	117.67 ± 26.38	<0.001
Urea (mmol/L)	9.5 (5.7, 17.5)	9.0 (5.3, 19.3)	8.2 (5.7, 16.2)	11.6 (7.1, 20.3)	0.058
Albumin (g/L)	40.7 (36.1, 44.5)	41.4 (35.9, 44.9)	41.4 (36.3, 44.4)	40.5 (35.4, 44.3)	0.876
Serum creatinine (µmol/L)	124 (78, 429)	113 (76, 531)	111 (78, 333)	174 (87, 448)	0.198
eGFR (mL/min/1.73 m²)	49.37 (10.98, 90.45)	60.71 (10.09, 103.38)	58.75 (16.55, 88.83)	26.21 (9.19, 76.33)	0.007
Uric acid (µmol/L)	350 (281, 423)	366 (284, 434)	343 (281, 405)	324 (275, 416)	0.357
Calcium (mmol/L)	2.24 (2.12, 2.33)	2.23 (2.11, 2.33)	2.24 (2.13, 2.33)	2.25 (2.11, 2.36)	0.800
Phosphorus (mmol/L)	1.21 (1.05, 1.42)	1.25 (1.05, 1.48)	1.17 (1.04, 1.37)	1.27 (1.07, 1.53)	0.173
Triglycerides (mmol/L)	1.37 (1.03, 1.97)	1.33 (1.00, 1.98)	1.43 (1.08, 2.18)	1.33 (1.01, 1.75)	0.505
Low-density lipoprotein cholesterol (mmol/L)	2.37 (1.71, 3.09)	2.25 (1.71, 2.99)	2.48 (1.73, 3.09)	2.41 (1.52, 3.30)	0.630
High-density lipoprotein cholesterol (mmol/L)	1.08 (0.86, 1.34)	1.06 (0.83, 1.31)	1.12 (0.89, 1.35)	1.05 (0.89, 1.35)	0.663
Total cholesterol (mmol/L)	4.05 (3.27, 5.05)	3.97 (3.25, 4.97)	4.08 (3.42, 5.30)	4.21 (3.15, 5.37)	0.280
QCT-derived and CT-based body composition parameters					
L3-SMA (cm²)	128.52 ± 32.38	135.87 ± 33.50	129.94 ± 31.16	115.15 ± 28.23	<0.001
L3-SMI (cm²/m²)	45.74 ± 9.89	47.89 ± 10.18	45.71 ± 9.90	42.45 ± 8.53	<0.001
IMAT (cm²)	17.1 (13.7, 21.8)	15.3 (12.4, 19.0)	17.3 (13.7, 21.7)	20.3 (15.8, 30.3)	<0.001
SAT (cm²)	119.2 (84.1, 159.2)	115.9 (79.9, 147.3)	121.2 (88.4, 157.4)	125.3 (89.5, 177.1)	0.266
VAT (cm²)	178.8 (122.3, 243.9)	174.8 (113.1, 235.1)	175.4 (125.8, 242.7)	189.8 (145.3, 250.8)	0.246
V/S ratio	1.53 (1.10, 2.10)	1.52 (1.09, 2.07)	1.60 (1.07, 2.00)	1.47 (1.16, 2.17)	0.976
Mean liver fat fraction (%)	8.13 (5.73, 11.10)	8.43 (6.07, 11.10)	7.47 (5.43, 11.00)	7.70 (5.77, 11.27)	0.711
Mean pancreatic fat fraction (%)	10.93 (8.40, 14.17)	10.07 (7.80, 12.70)	10.77 (8.40, 14.13)	12.80 (10.10, 16.70)	<0.001

*Note*: Continuous variables were presented as mean ± standard deviation (SD) or median (interquartile range [IQR]) according to their distribution. Categorical variables were presented as n (%). Comparisons among groups were performed using one-way analysis of variance (ANOVA), the Kruskal–Wallis H test, Pearson’s chi-square test, or Fisher’s exact test, as appropriate.

Abbreviations: BMI, body mass index; CKD, chronic kidney disease; eGFR, estimated glomerular filtration rate; L3-SMA, L3 skeletal muscle area; L3-SMI, L3 skeletal muscle index; IMAT, intermuscular adipose tissue; SAT, subcutaneous adipose tissue; VAT, visceral adipose tissue; V/S ratio, visceral-to-subcutaneous fat ratio.

Among the 40 randomly selected patients, mean pancreatic fat fraction showed good interobserver reproducibility (ICC = 0.860, 95% CI: 0.715–0.929).

### Association between mean pancreatic fat fraction and bone mass status

Ordinal logistic regression analysis showed that each 5-percentage-point increase in mean pancreatic fat fraction was associated with higher odds of being in a more severe bone mass category in the crude model (OR = 1.520, 95% CI: 1.221–1.892, *p* < 0.001). In the fully adjusted model (Model 3), the association remained statistically significant after adjustment for age, sex, BMI, eGFR, diabetes mellitus, hypertension, and L3-SMI (OR = 1.481, 95% CI: 1.152–1.905, *p* = 0.002). Detailed results are presented in Table 2. There was no significant correlation between mean pancreatic fat fraction and L3-SMI (Spearman’s ρ = 0.074, *p* = 0.180). Furthermore, no evidence of problematic multicollinearity was observed in the multivariable models, with all VIFs < 2.0. The overall Brant test for the fully adjusted model was not statistically significant (χ^2^ = 13.23, df = 8, *p* = 0.100), indicating no evidence of violation of the proportional odds assumption.

**Table 2.** Ordinal logistic regression analysis of the association between mean pancreatic fat fraction and bone mass status.

Variable	Model	OR	95% CI	P value
Mean pancreatic fat fraction	Crude model	1.520	1.221–1.892	<0.001
	Model 1	1.452	1.141–1.849	0.002
	Model 2	1.481	1.151–1.905	0.002
	Model 3	1.481	1.152–1.905	0.002

*Note*: OR represents the odds ratio associated with each 5-percentage-point increase in mean pancreatic fat fraction. Crude model: unadjusted; Model 1: adjusted for age and sex; Model 2: additionally adjusted for BMI, eGFR, diabetes mellitus, and hypertension; Model 3: additionally adjusted for L3-SMI based on Model 2.

Abbreviations: OR, odds ratio; CI, confidence interval; BMI, body mass index; eGFR, estimated glomerular filtration rate; L3-SMI, L3 skeletal muscle index.

### Dose–response relationship between mean pancreatic fat fraction and bone mass status

RCS analysis showed a significant overall association between mean pancreatic fat fraction and more severe bone mass categories after adjustment for age, sex, BMI, eGFR, diabetes mellitus, hypertension, and L3-SMI (P for overall = 0.009). However, there was no evidence of a nonlinear association (P for nonlinearity = 0.290), suggesting that the association was approximately linear (Figure 3).

**Figure 3.**
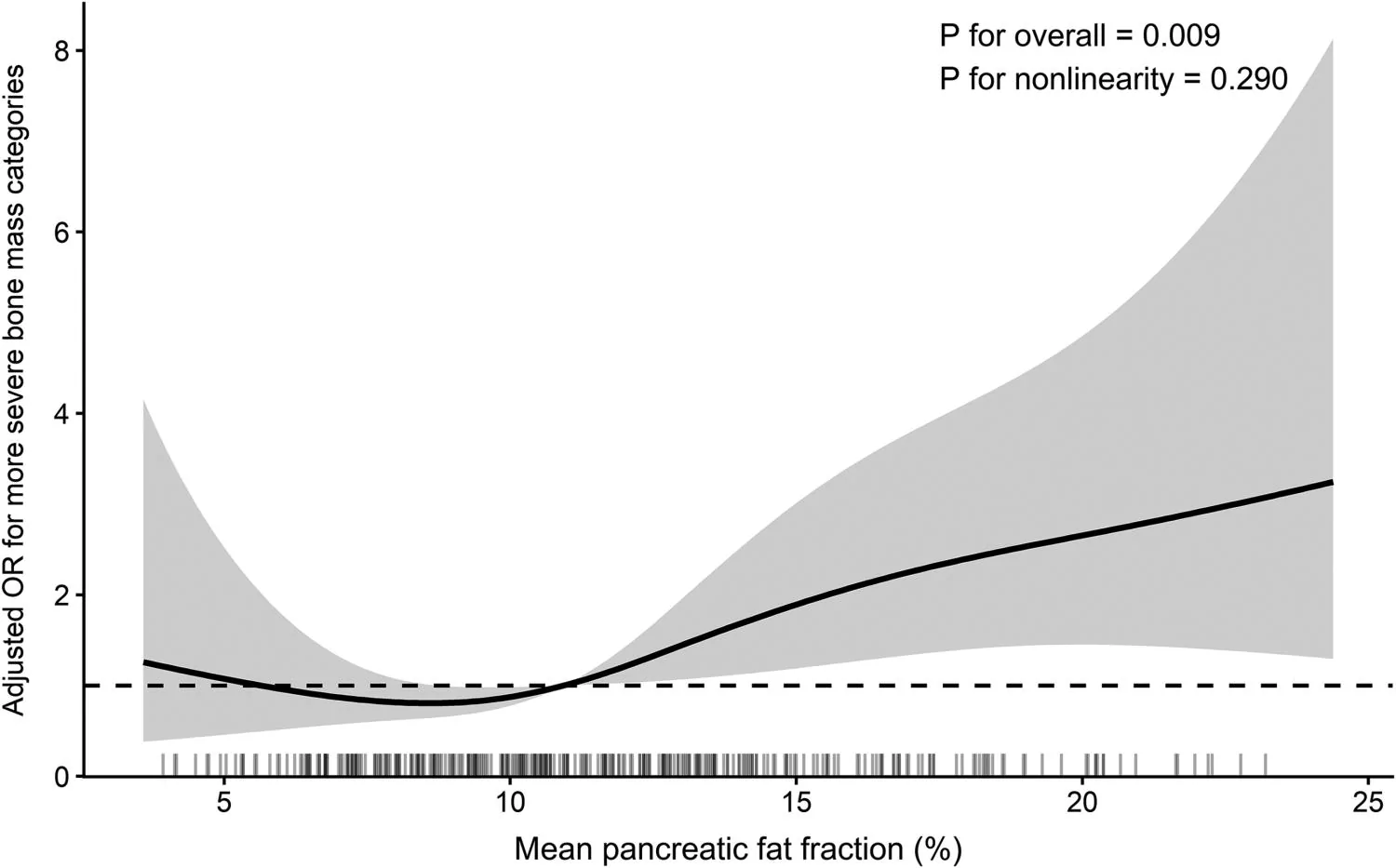
Restricted cubic spline (RCS) analysis of the association between mean pancreatic fat fraction and bone mass status. RCS analysis was performed to evaluate the dose–response relationship between mean pancreatic fat fraction and the odds of being in a more severe bone mass category. The model was adjusted for age, sex, BMI, eGFR, diabetes mellitus, hypertension, and L3-SMI, with four knots used for the spline function. The solid line represents the adjusted OR, the shaded area represents the 95% CI, and the horizontal dashed line indicates an OR of 1. The model was fitted using data from all 327 patients. For visualization purposes, the x-axis displays the 1st–99th percentile range of mean pancreatic fat fraction.

### Subgroup analysis

To further evaluate the consistency of the association between mean pancreatic fat fraction and the odds of being in a more severe bone mass category across clinical subgroups, subgroup analyses were performed according to sex, age, diabetes status, and CKD stage. The effect estimates were generally consistent across subgroups. Interaction analyses showed no statistically significant interactions for sex, age, diabetes status, or CKD stage (P for interaction = 0.837, 0.796, 0.886, and 0.222, respectively) (Figure 4).

**Figure 4.**
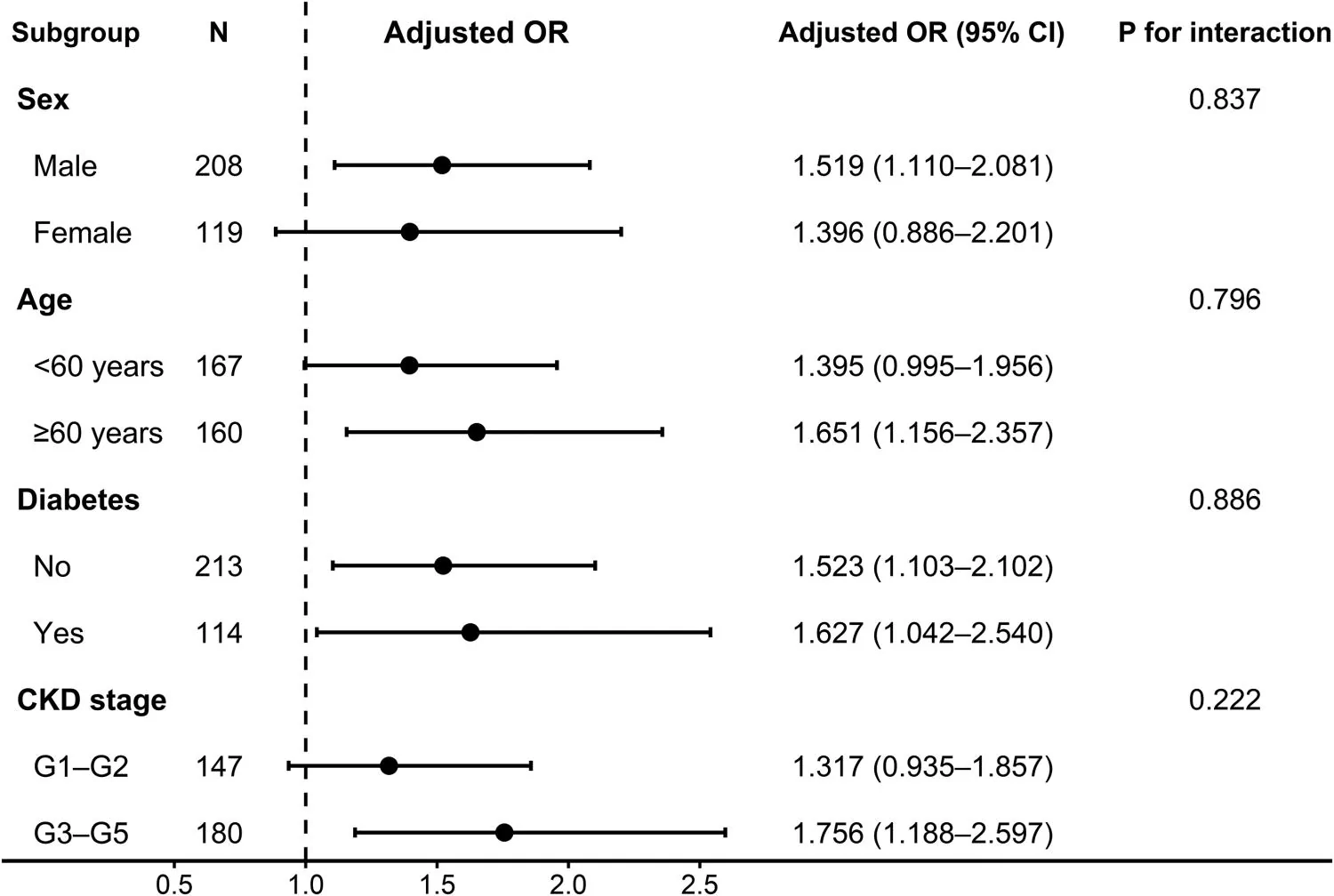
Subgroup analysis of the association between mean pancreatic fat fraction and the odds of being in a more severe bone mass category. Subgroup analyses were performed according to sex, age, diabetes status, and CKD stage. The points represent adjusted ORs for the odds of being in a more severe bone mass category associated with each 5-percentage-point increase in mean pancreatic fat fraction, and the horizontal lines represent 95% CIs. All subgroup models were adjusted for age, sex, BMI, eGFR, diabetes mellitus, hypertension, and L3-SMI, except for the corresponding stratification variable. In the CKD stage subgroup analysis, eGFR was not additionally adjusted. The vertical dashed line indicates an OR of 1. P for interaction was used to assess potential effect modification by each stratification factor.

### Sensitivity analysis

After excluding 51 patients receiving dialysis, 276 non-dialysis patients remained for the sensitivity analysis. The distribution of clinical and imaging characteristics across bone mass categories was generally consistent with that observed in the overall population (Supplementary Table S1). In the fully adjusted model (Model 3), each 5-percentage-point increase in mean pancreatic fat fraction remained significantly associated with higher odds of being in a more severe bone mass category (OR = 1.521, 95% CI: 1.164–1.988, *p* = 0.002; Supplementary Table S2), supporting the robustness of the primary findings.

## Discussion

In this study, we used QCT-based assessments to characterize bone mass status and body composition in patients with CKD and primarily investigated the association between pancreatic fat fraction and bone mass status. Mean pancreatic fat fraction increased progressively across worsening bone mass categories. After adjustment for the measured covariates, higher mean pancreatic fat fraction remained associated with more severe bone mass categories, with consistent findings across progressively adjusted models. RCS analysis demonstrated a significant overall association, with no evidence of a nonlinear relationship. Importantly, the association remained significant after excluding patients receiving dialysis, with an effect estimate comparable to that observed in the primary analysis, supporting the robustness of the main findings. Furthermore, subgroup analyses showed generally consistent effect estimates across sex, age, diabetes status, and CKD stage, with no statistically significant interactions detected. Collectively, these findings suggest an association between higher pancreatic fat fraction and more severe bone mass abnormalities in patients with CKD.

Ectopic fat deposition is increasingly recognized as an important phenotype of impaired adipose tissue storage capacity and systemic metabolic dysregulation rather than simply a consequence of excessive lipid accumulation in non-adipose tissues [17]. Accumulating evidence indicates that ectopic fat deposition at different anatomical sites may be associated with reduced bone mass and impaired bone microarchitecture [18]. However, the relationship between pancreatic fat and skeletal health remains poorly understood. Previous studies have reported that greater pancreatic fat infiltration is associated with a higher risk of vertebral fractures in patients with type 2 diabetes mellitus, whereas higher pancreatic fat content has been independently associated with greater bone marrow fat content in individuals with obesity [19,20]. In addition, a large population-based study found an independent association between greater pancreatic fat deposition and impaired renal function [21]. These findings point to potential links between pancreatic fat, skeletal health, and kidney function. Nevertheless, evidence specifically addressing the association between pancreatic fat and bone mass status in patients with CKD remains scarce.

CKD patients are at high risk of bone loss and fractures, reflecting complex disturbances in mineral metabolism and endocrine regulation. As kidney function declines, abnormalities in phosphate metabolism, the vitamin D–parathyroid hormone (PTH) axis, and inflammatory status may develop, potentially affecting bone turnover, mineralization, and bone strength [22,23]. Beyond traditional abnormalities in mineral metabolism, growing attention has focused on the relationship between body composition and bone mass in patients with CKD. Previous studies have shown that body composition is associated with BMD changes in patients with non-dialysis CKD, with greater lean tissue mass associated with less BMD loss [5]. In our study, the association between mean pancreatic fat fraction and bone mass status remained significant after adjustment for renal function and skeletal muscle mass. This finding suggests that the association between pancreatic fat and bone mass status may not be fully explained by renal dysfunction or muscle loss, providing additional imaging evidence linking ectopic fat with bone mass abnormalities in patients with CKD.

The association between pancreatic fat and abnormal bone mass status may involve several metabolic pathways. One possible mechanism is impaired glucose metabolism. Pancreatic fat deposition has been associated with glucose dysregulation and altered β-cell function [24]. Insulin-like growth factor-1 (IGF-1) signaling also plays an important role in osteoblast function and bone formation [25], suggesting a potential link between metabolic abnormalities associated with pancreatic fat deposition and altered bone remodeling. Inflammation may provide another link between pancreatic fat and skeletal health. Pancreatic ectopic fat deposition has been associated with adipokine dysregulation and chronic low-grade inflammation [26]. Inflammatory mediators, including tumor necrosis factor-α (TNF-α) and interleukin-6 (IL-6), can influence osteoblast and osteoclast activity and contribute to disturbances in bone remodeling [27]. CKD itself is also characterized by persistent inflammation, which has been linked to abnormalities in bone metabolism [28]. Impaired adipose tissue storage capacity may also promote the redistribution of excess lipids to non-adipose organs, including the pancreas, liver, and skeletal muscle, resulting in ectopic fat accumulation and lipotoxicity [29]. Therefore, a higher pancreatic fat fraction may reflect not only local lipid accumulation but also systemic lipid redistribution and metabolic dysregulation, potentially linking metabolic and inflammatory abnormalities to abnormal bone mass status. However, insulin resistance, inflammatory biomarkers, and markers of lipotoxicity were not assessed in this study. Whether these pathways mediate the association between pancreatic fat and abnormal bone mass status therefore requires further investigation.

Further analysis showed no evidence of a nonlinear association between mean pancreatic fat fraction and bone mass status, while the direction of the effect estimates was generally consistent across clinical subgroups. No statistically significant interaction was detected for sex, age, diabetes status, or CKD stage. However, these findings should be interpreted cautiously because the moderate sample sizes and wide confidence intervals in some subgroups indicate limited statistical power to detect potential effect modification. Therefore, the absence of statistically significant interactions should not be interpreted as definitive evidence of no subgroup differences, and larger studies are needed to evaluate potential effect modification more reliably.

Our findings suggest that pancreatic fat may provide information on bone mass abnormalities in patients with CKD beyond that provided by renal function and conventional body composition parameters. QCT allows simultaneous quantitative assessment of vertebral bone mass, skeletal muscle, and adipose tissue using clinically acquired CT images, without the need for additional imaging examinations. In this study, the association between pancreatic fat fraction and bone mass status remained significant after adjustment for renal function, BMI, and skeletal muscle mass. These findings support the potential value of incorporating pancreatic fat assessment into QCT-based body composition analysis and may help characterize metabolic features associated with bone health in patients with CKD.

Several limitations of this study should be acknowledged. First, this was a single-center cross-sectional study; therefore, the temporal and causal relationships between pancreatic fat deposition and abnormal bone mass status cannot be established. In addition, because of the retrospective design, no *a priori* sample-size calculation was performed, which may have limited the statistical power of some analyses. Multicenter prospective studies are needed to confirm these findings. Second, although multiple potential confounding factors were considered, residual or unmeasured confounding cannot be excluded, particularly from factors such as parathyroid hormone levels, 25-hydroxyvitamin D concentrations, bone turnover markers, medication use, and lifestyle factors. Third, although blood samples were collected before the first dialysis session during the index hospitalization, laboratory parameters in patients receiving dialysis may have been influenced by the timing of previous dialysis sessions. Finally, the mean pancreatic fat fraction was estimated by averaging ROI-based measurements from the pancreatic head, body, and tail. Although this approach showed good interobserver reproducibility in our study, intraobserver reproducibility was not assessed. Selected ROI-based measurements may not fully capture regional heterogeneity in pancreatic fat distribution. Whole-pancreas volumetric quantification may provide a more comprehensive assessment of pancreatic fat and should be considered in future studies.

## Conclusion

In conclusion, higher mean pancreatic fat fraction was associated with more severe bone mass categories in patients with CKD after adjustment for the measured covariates. Pancreatic fat may represent a metabolic phenotype associated with bone mass abnormalities and may provide additional information for QCT-based assessment of skeletal health in patients with CKD.

## Supplementary Material

Supplementary_Material_R2.docx

## Data Availability

The data that support the findings of this study are available from the corresponding author upon reasonable request, subject to applicable ethical and institutional restrictions. The data are not publicly available due to patient privacy considerations.
