# Supplementary material for: Association between pancreatic ectopic fat deposition and QCT-assessed bone mass status in patients with chronic kidney disease: a cross-sectional study
Source: Ren Fail. 2026 Nov 2;48(1):2749918. doi: 10.1080/0886022X.2026.2749918 (PMC13729618; doi:10.1080/0886022X.2026.2749918)
Supplement: Supplementary file 1 [file IRNF_A_2749918_SM9666.docx]

**Supplementary_Material**

**Supplementary Table S1. Clinical and imaging characteristics of CKD patients according to bone mass status after excluding patients receiving dialysis.**

| **Characteristics** | **Overall (N = 276)** | **Normal bone mass (N = 108)** | **Osteopenia (N = 102)** | **Osteoporosis (N = 66)** | **P value** |
| --- | --- | --- | --- | --- | --- |
| **Demographic and clinical characteristics** | | | | | |
| Sex, n (%) | | | | | 0.003 |
| Male | 167 (60.5) | 72 (66.7) | 67 (65.7) | 28 (42.4) |  |
| Female | 109 (39.5) | 36 (33.3) | 35 (34.3) | 38 (57.6) |  |
| Age (years) | 57.77 ± 13.54 | 47.40 ± 12.36 | 61.67 ± 9.17 | 68.71 ± 8.32 | <0.001 |
| BMI (kg/m²) | 24.22 (21.91, 26.12) | 24.29 (21.79, 27.11) | 23.92 (21.91, 26.06) | 24.22 (21.97, 25.78) | 0.442 |
| Hypertension, n (%) | | | | | 0.059 |
| No | 106 (38.4) | 49 (45.4) | 39 (38.2) | 18 (27.3) |  |
| Yes | 170 (61.6) | 59 (54.6) | 63 (61.8) | 48 (72.7) |  |
| Diabetes mellitus, n (%) | | | | | <0.001 |
| No | 184 (66.7) | 87 (80.6) | 58 (56.9) | 39 (59.1) |  |
| Yes | 92 (33.3) | 21 (19.4) | 44 (43.1) | 27 (40.9) |  |
| CKD stage, n (%) | | | | | 0.009 |
| G1 | 81 (29.3) | 44 (40.7) | 28 (27.5) | 9 (13.6) |  |
| G2 | 66 (23.9) | 21 (19.4) | 28 (27.5) | 17 (25.8) |  |
| G3 | 49 (17.8) | 15 (13.9) | 21 (20.6) | 13 (19.7) |  |
| G4 | 36 (13.0) | 10 (9.3) | 11 (10.8) | 15 (22.7) |  |
| G5 | 44 (15.9) | 18 (16.7) | 14 (13.7) | 12 (18.2) |  |
| **Laboratory parameters** | | | | | |
| Hb (g/L) | 133.16 ± 28.20 | 137.31 ± 29.96 | 134.57 ± 27.83 | 124.21 ± 23.89 | 0.009 |
| Urea (mmol/L) | 8.0 (5.4, 14.8) | 6.6 (5.1, 13.7) | 7.45 (5.6, 14.7) | 9.55 (6.8, 15.6) | 0.099 |
| ALB (g/L) | 41.50 (35.85, 44.80) | 41.70 (35.85, 45.40) | 41.50 (36.10, 44.40) | 40.75 (34.60, 44.70) | 0.861 |
| Scr (µmol/L) | 102 (75, 237.5) | 92.5 (69, 226.5) | 96.5 (77, 204) | 128.5 (77, 250) | 0.283 |
| eGFR (mL/min/1.73 m²) | 65.29 (23.23, 96.55) | 81.38 (29.52, 106.18) | 66.47 (30.05, 91.55) | 46.63 (19.77, 81.01) | 0.002 |
| UA (µmol/L) | 357 (287.5, 433) | 369 (284.5, 453) | 349 (293, 410) | 331.5 (290, 426) | 0.490 |
| Ca (mmol/L) | 2.23 (2.12, 2.33) | 2.25 (2.13, 2.32) | 2.23 (2.12, 2.32) | 2.24 (2.10, 2.34) | 0.917 |
| P (mmol/L) | 1.18 (1.04, 1.38) | 1.18 (1.01, 1.40) | 1.16 (1.04, 1.34) | 1.24 (1.06, 1.40) | 0.659 |
| TG (mmol/L) | 1.41 (1.05, 2.01) | 1.37 (1.04, 2.02) | 1.42 (1.08, 2.23) | 1.38 (1.02, 1.82) | 0.848 |
| LDL-C (mmol/L) | 2.51 (1.77, 3.24) | 2.42 (1.83, 3.09) | 2.57 (1.74, 3.24) | 2.52 (1.77, 3.40) | 0.636 |
| HDL-C (mmol/L) | 1.12 (0.87, 1.37) | 1.11 (0.84, 1.35) | 1.14 (0.89, 1.37) | 1.10 (0.90, 1.36) | 0.809 |
| CHOL (mmol/L) | 4.33 (3.47, 5.29) | 4.15 (3.49, 5.04) | 4.36 (3.46, 5.40) | 4.42 (3.37, 5.41) | 0.641 |
| **QCT-derived and CT-based body composition parameters** | | | | | |
| L3-SMA (cm²) | 128.63 ± 32.82 | 135.63 ± 35.46 | 129.40 ± 31.48 | 115.99 ± 26.55 | <0.001 |
| L3-SMI (cm²/m²) | 45.89 ± 10.03 | 48.06 ± 10.60 | 45.53 ± 9.99 | 42.90 ± 8.25 | 0.004 |
| IMAT (cm²) | 16.95 (13.35, 21.40) | 15.10 (12.25, 18.85) | 17.30 (13.60, 21.70) | 19.55 (15.80, 28.90) | <0.001 |
| SAT (cm²) | 121.05 (89.65, 161.35) | 116.95 (81.60, 157.75) | 123.65 (92.70, 157.40) | 129.60 (95.90, 183.40) | 0.264 |
| VAT (cm²) | 179.35 (121.10, 243.65) | 174.50 (99.80, 235.05) | 174.70 (125.80, 240.50) | 200.40 (148.10, 252.00) | 0.109 |
| V/S ratio | 1.48 (1.04, 2.01) | 1.43 (0.98, 2.00) | 1.56 (1.07, 1.96) | 1.48 (1.11, 2.13) | 0.669 |
| Mean liver fat fraction (%) | 7.73 (5.63, 10.85) | 8.18 (5.93, 10.90) | 7.45 (5.33, 10.83) | 7.67 (5.73, 10.87) | 0.713 |
| Mean pancreatic fat fraction (%) | 11.00 (8.37, 14.27) | 10.02 (7.82, 12.72) | 11.00 (8.27, 14.50) | 13.35 (10.20, 16.70) | <0.001 |

**Note:** Continuous variables were presented as mean ± standard deviation (SD) or median (interquartile range [IQR]) according to their distribution. Categorical variables were presented as n (%). Comparisons among groups were performed using one-way analysis of variance (ANOVA), the Kruskal–Wallis H test, Pearson’s chi-square test, or Fisher’s exact test, as appropriate.

**Abbreviations:** BMI, body mass index; CKD, chronic kidney disease; Hb, hemoglobin; ALB, albumin; Scr, serum creatinine; eGFR, estimated glomerular filtration rate; UA, uric acid; Ca, serum calcium; P, serum phosphorus; TG, triglycerides; LDL-C, low-density lipoprotein cholesterol; HDL-C, high-density lipoprotein cholesterol; CHOL, total cholesterol; L3-SMA, L3 skeletal muscle area; L3-SMI, L3 skeletal muscle index; IMAT, intermuscular adipose tissue; SAT, subcutaneous adipose tissue; VAT, visceral adipose tissue; V/S ratio, visceral-to-subcutaneous fat ratio.

**Supplementary Table S2. Sensitivity analysis of the association between mean pancreatic fat fraction and bone mass status after excluding patients receiving dialysis.**

| Variable | Model | OR | 95% CI | P value |
| --- | --- | --- | --- | --- |
| Mean pancreatic fat fraction | Crude model | 1.530 | 1.211–1.934 | <0.001 |
|  | Model 1 | 1.486 | 1.149–1.921 | 0.003 |
|  | Model 2 | 1.521 | 1.164–1.987 | 0.002 |
|  | Model 3 | 1.521 | 1.164–1.988 | 0.002 |

**Note:** OR represents the odds ratio associated with each 5-percentage-point increase in mean pancreatic fat fraction. Crude model: unadjusted; Model 1: adjusted for age and sex; Model 2: additionally adjusted for BMI, eGFR, diabetes mellitus, and hypertension; Model 3: additionally adjusted for L3-SMI based on Model 2.

**Abbreviations:** OR, odds ratio; CI, confidence interval; BMI, body mass index; eGFR, estimated glomerular filtration rate; L3-SMI, L3 skeletal muscle index.
